# Comparative assessment of the reaming characteristics of two different technologies for intramedullary bone graft harvesting

**DOI:** 10.1007/s00402-026-06270-y

**Published:** 2026-04-09

**Authors:** Simone Guttau, Merle Lange, Ronja Finze, Claudia Beimel, Siamak Saifzadeh, Jonathan Gospos, Marie-Luise Wille, Philipp Kobbe, Markus Laubach

**Affiliations:** 1 Stryker Trauma GmbH, Schönkirchen, Germany; 2 Department for Trauma and Reconstructive Surgery, University Hospital of the Martin Luther University Halle, Halle (Saale), Germany; 3 Department of Hand, Plastic and Reconstructive Surgery, BG Trauma Center Ludwigshafen, Heidelberg University, Ludwigshafen, Germany; 4 Australian Research Council (ARC) Training Centre for Multiscale 3D Imaging, Modelling, and Manufacturing (M3D Innovation), Queensland University of Technology, Queensland 4000 Brisbane, Australia; 5 Department for Trauma and Reconstructive Surgery, BG Hospital Bergmannstrost Halle, Halle (Saale), Germany; 6 Department of Orthopaedics and Trauma Surgery, Musculoskeletal University Center Munich (MUM), LMU University Hospital, LMU Munich, Munich, Germany; 7 Medical Engineering Research Facility, Queensland University of Technology, Queensland 4032 Brisbane, Australia; 8 Max Planck Queensland Centre (MPQC) for the Materials Science of Extracellular Matrices, Queensland University of Technology (QUT), Queensland 4000 Brisbane, Australia; 9 Centre for Biomedical Technologies, School of Mechanical, Medical and Process Engineering, Queensland University of Technology, Queensland 4000 Brisbane, Australia; 10 Centre for Biomedical Technologies, School of Biomedical Sciences, Queensland University of Technology, Queensland 4000 Brisbane, Australia

**Keywords:** Intramedullary reaming, RIA 2 system, ARA concept, *in vivo*

## Abstract

**Introduction:**

The study aimed to compare the biomechanical impact and reaming performance of two intramedullary bone graft harvesting techniques: the established Reamer-Irrigator-Aspirator 2 (RIA 2) system and a novel aspirator+reaming-aspiration (ARA) concept.

**Materials and methods:**

In a preclinical in vivo sheep model, sixteen femora were assigned to either the RIA 2 or ARA group (*n* = 8 each). Biomechanical testing, computed tomography (CT)-based 3D bone geometry analysis, and fracture line assessment were performed postoperatively. Bending stiffness of the reamer shafts, cortical wall thickness pre- and post-reaming, and torsional stability of reamed femora were evaluated. Statistical analysis included TOST, Mann-Whitney U, and Shapiro-Wilk tests, considering values of *p* < 0.05 as statistically significant.

**Results:**

Both reaming systems produced the greatest cortical thinning medially in the midsections of the analyzed region. No statistically significant differences were observed in postoperative cortical wall thickness, bone removal rates, or torsional stability between groups (all *p* > 0.05). Bending stiffness was significantly higher for the RIA 2 system (*p* = 0.004). In both groups, fractures predominantly initiated medially or at sites of previous drill holes. The ARA concept demonstrated equivalent biomechanical and reaming performance compared to the RIA 2 system, with a comparable safety profile.

**Conclusions:**

In this preclinical in vivo comparison, the RIA 2 system and the ARA concept with BixCut reamers demonstrated equivalent reaming performance regarding cortical bone removal and femoral shaft geometry. Although the RIA 2 drive shaft exhibited greater bending stiffness, both systems induced comparable medial cortical thinning in the mid-diaphyseal region. Three-dimensional geometric analysis and ex vivo biomechanical testing showed no significant differences in residual cortical thickness or torsional stability. Fracture patterns were similar and correlated with areas of maximal medial thinning. Overall, both systems provided comparable reaming functionality and biomechanical outcomes.

**Supplementary Information:**

The online version contains supplementary material available at 10.1007/s00402-026-06270-y.

## Introduction

Impaired fracture healing, bony defects following tumor resection, and posttraumatic bone defects have contributed to a growing demand for autologous bone graft harvesting. Bone graft harvested from the intramedullary canal of long bones has been associated with relevant bone regenerative capacity [1–3]. The Reamer-Irrigator-Aspirator (RIA) 2 system (DePuy Synthes) allows for harvesting of significant volumes of autologous bone graft from the intramedullary canal of the femur or tibia [4]. Good quality of the bone graft and low complication risks have been reported when harvesting with this device [5, 6]. Despite this, some reports describe the risk of blood loss and complications due to aggressive and eccentric reaming, especially with its application outside of so-called “centers of excellence” [7].

Recently, the aspirator+reaming-aspiration (ARA) concept has been introduced as a potentially more intuitive alternative for intramedullary bone graft harvesting [8]. Building upon the alternative concept an innovative aspirator prototype device first applies to remove bone marrow, followed by using standard intramedullary reamers and the aspirator for harvesting of morselized endosteal bone chips [8, 9]. In contrast to the RIA 2 system, the ARA concept does not require irrigation fluid during the harvesting process and can be used with standard intramedullary reamers that are very common among surgeons. Preclinical data suggest that the ARA concept is a promising alternative to the RIA 2 system for intramedullary bone graft harvesting, offering a comparable safety profile [10, 11] and yielding bone grafts with strong osteogenic regenerative potential [8, 12].

Moreover, Gehweiler et al. (2021) [13] observed that using the RIA technique was associated with predilection sites for cortical thickness reduction after reaming. Therefore, in case new concepts for intramedullary bone graft harvesting, such as the ARA concept, investigation of the three-dimensional (3D) geometry of the reamed bone as a function of the reaming diameter and its influence on the biomechanical stability is required.

Thus, in this large animal in vivo study comparing the RIA 2 system (RIA 2 group) and the ARA concept (ARA group), the following were investigated: (1) mechanical properties of the reaming systems, (2) the effect of reaming on 3D cortical bone geometry, (3) the biomechanical stability of the reamed sheep femora, and (4) fracture line patterns.

## Materials and methods

The study design first included a mechanical examination of the RIA 2 system and standard reamers. Further on, a large animal (sheep) study was conducted to assess the reaming effect on 3D cortical bone geometry, biomechanical stability of the reamed femora as well as fracture line evaluation. The respective methods are described hereafter.

### Mechanical bending testing RIA 2 system and standard reamers

Bending stiffness testing was performed with the drive shaft for RIA 2 system and the BixCut system (Stryker). Reamer shafts were centrally mounted in a load cell (Schenk PSA 10) equipped with a 4-point bending fixture, using a support span distance of 114 mm and a loading span of 38 mm (Supplementary Fig. 1).

Bending under axial displacement with a target speed of 0.2 mm/sec was performed until a displacement of 8 mm was reached. Bending load was recorded, and the bending stiffness K was calculated for every test device using preset load points at 50 N and 200 N bending load as

1 $$K=\frac{\varDelta F}{\varDelta h}$$

where ΔF is the difference in applied force and Δh represents the deflection height.

### Assessment of reaming effect on 3D cortical bone geometry, biomechanical stability of the reamed sheep femora, and fracture line

#### In vivo testing

Ethical approval for this study was obtained from the Queensland University of Technology Animal Ethics Committee (Ethics Approval Number 2000000593). The comprehensive ex vivo examination of sheep femora presented in this work have been carried out as part of a secondary analysis, while the results of the primary in vivo study have been published elsewhere [9–11]. All in vivo experiments were conducted at the QUT Medical Engineering Research Facility (MERF), located on the Prince Charles Hospital campus in Chermside, Queensland, Australia. Sheep were sourced from a local agricultural supplier, and each animal underwent a comprehensive preoperative veterinary assessment in accordance with an established and previously validated health screening protocol [14]. All procedures were performed in compliance with the Australian Code for the Care and Use of Animals for Scientific Purposes. Study design, conduct, and reporting adhered to the ARRIVE 2.0 (Animal Research: Reporting of In Vivo Experiments) guidelines to ensure methodological rigor and transparency [15].

Sixteen sheep were randomly allocated to two groups of RIA 2 and ARA, each consisting of 8 animals. Group allocation of sheep was performed following the smallest medullary diameter of the femoral shaft using computed tomography (CT) images to achieve matching of sheep in both groups with similar intramedullary diameters, as described in detail [9]. For the study, female Merino sheep with a body weight of 41–51 kg and 1–2 years of age were used and subjected to intramedullary bone graft harvesting either applying the RIA 2 system or the ARA concept [9].

For in-depth details on the positioning of the sheep, the surgical approach, and the reaming technique utilized, please refer to previous literature, including detailed study protocol [9–11]. In brief, sheep were placed in right lateral recumbency, and surgical site preparation was performed as previously described [14], ensuring access to the left proximal femur via an anterograde approach. In each sheep only the left femur was operated. All procedures were conducted by the same surgeon (Ma.L.) under consistent surgical conditions. The femur was flexed at 90° in the hip joint, and a longitudinal skin incision was made 1–2 cm proximal to the palpable greater trochanter. The biceps femoris was incised, the iliacus tendon identified and severed, and tissue over the trochanteric fossa removed using a periosteal elevator. Under X-ray guidance, a K-wire was inserted, followed by a Ø10 mm cannulated reamer (Stryker) to access the medullary canal, and a ball tip guide wire was positioned. The canal opening was enlarged with an Ø11 mm BixCut fixed-head reamer (Stryker). Autologous bone graft was harvested using either the RIA 2 system or the ARA concept. The RIA 2 system integrates irrigation and aspiration in a single-step reaming process. For both harvesting system, reaming began with a head 2 mm narrower than the femoral isthmus diameter, increased by 1 mm increments for the first two steps, then by 0.5 mm increments until X-rays showed ~ 0.5–1 mm residual cortex. In the ARA group, bone marrow was first aspirated using a prototype device, followed by reaming using the BixCut reamer in the same incremental steps. After each reaming, bone chips were collected by introducing the aspirator nozzle into the canal with reciprocating movements, gently contacting the endocortex. For the RIA 2 system, reaming and bone graft harvesting was done in a single step. Upon completion of the experiments, the animals were euthanized while still under anesthesia with 160 mg/kg pentobarbital sodium (Lethabarb^®^ IV). Left and right legs were harvested and kept frozen at -20 °C for further analyses.

#### Three-dimensional geometry of cortical bone before and after harvesting

Computed tomography scans were performed from both legs (left and right) of each sheep. The in vivo CT images taken for presurgical sheep allocation and CT images after the surgical procedure were used to assess the effects on 3D cortical bone geometry. The resolution of all CT scans was 1.0 mm layer thickness with a increment of 0.5 mm. All CT data sets were exported as digital imaging and communications in medicine (DICOM) files for further analysis. Segmentation of the raw CT data (DICOMs) was conducted with the software Mimics (Materialise Interactive Medical Image Control System) Medical 23.0 (Materialise NV, Leuven, Belgium). The cortical bone models were segmented using a threshold value of 580 HU [16, 17]. All models were converted to STL format for further processing. Further modelling and analysis of the cortical bone models was conducted with Geomagic Control 2014.3.0 (3D Systems 2016). The receiver operating characteristic (ROC), as well as the femur length and the isthmus diameter prior reaming were determined. Three-dimensional comparison of the pre- and post-reaming bones was carried out at the middle 40% of the bone length, which represented the region of major reduction in the cortical thickness.

**Fig. 1:**
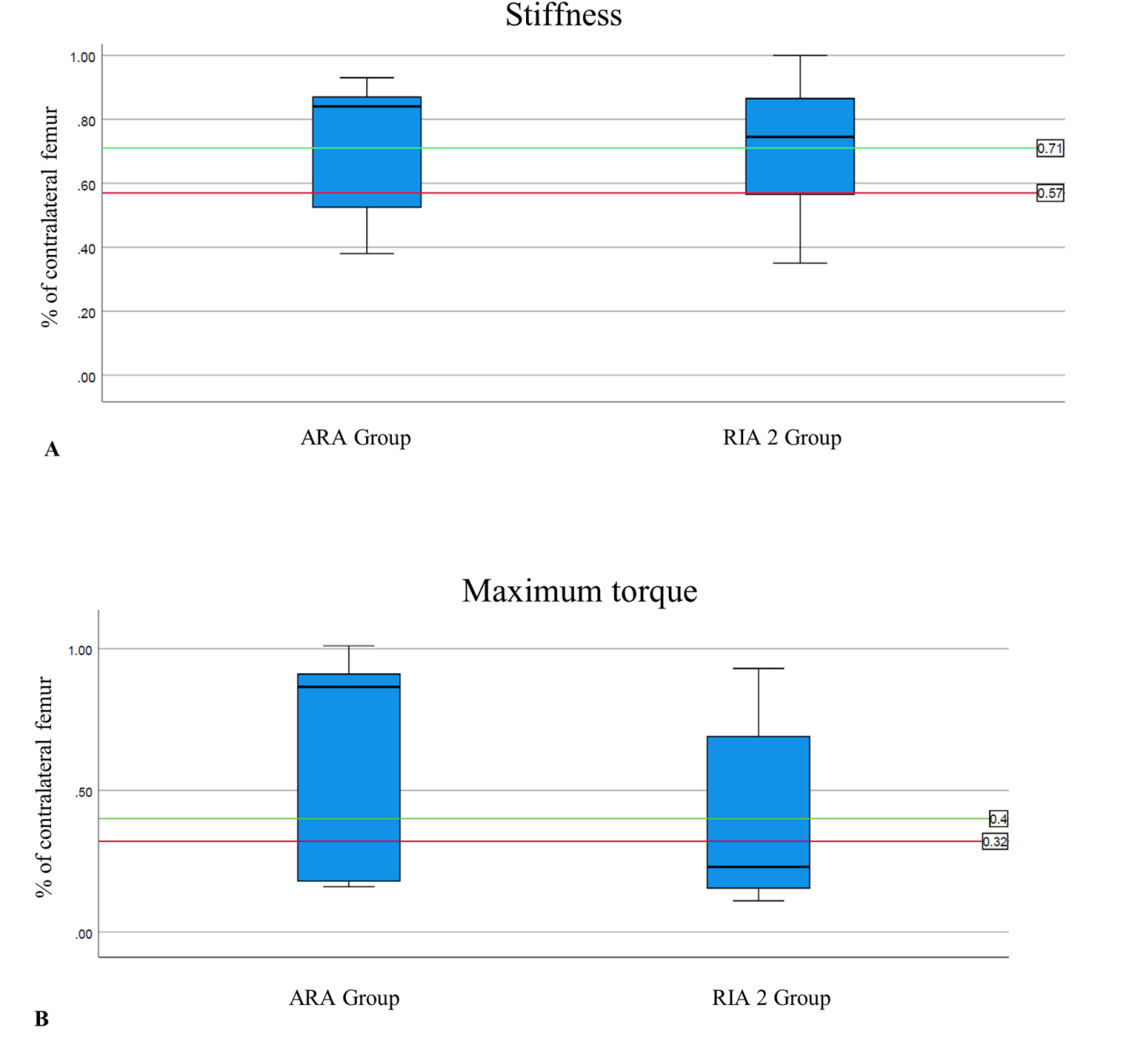
Exemplary visualization of the sections with resulting analytical points used in this study, left: sagittal bone cut, right: bone cut by frontal plane

Moreover, femora were equally divided into 8 parts, each representing steps of 5% of the cortical bone length. For this, 9 cross sections were defined rectangular to the longitudinal axis, and the section crossing the middle point of the long axis was defined as 0% plane. Additionally, sagittal and frontal planes were aligned manually to the bone shapes, resulting in 72 representative analytical points, 36 at the inner and 36 at the outer surfaces of the cortical bone (Fig. 1). The cortical wall thickness was defined as the distance between the inner and outer surface of the cortex and measured for all 9 sections. The section with the absolute lowest cortical wall thickness after reaming was defined for both groups. Additionally, the total decrease in wall thickness after reaming was analyzed for all sections.

#### Biomechanical testing of femora ex vivo

Post-mortem mechanical testing was performed according to a previously validated protocol [14, 18, 19]. After limb explantation, the non-operated and operated femora were separated from the hind limbs. Removal of the soft tissues from the femora and subsequent embedding of the proximal and distal femoral ends in custom-made jigs using Palapress embedding cement (Palapress vario dental acrilyc powder (cat# 64707785) and monomer (cat# 64707817); Kulzer GmbH, Hanau, Germany). After hardening, mounting of the specimens in a biaxial material testing machine (Instron 8874 Axial-Torsion System, Instron, Norwood, USA) was performed (test setup shown in Supplementary Fig. 2). Torsion under angular displacement with a target speed of 0.5˚/s and a constant compressive load of 0.05 kN was performed until the onset of fracture, indicated by a marked increase in displacement at which point the process was immediately stopped (exemplary test video: Supplementary Video 1). For the operated femur, the maximum torsional moment (TM) and torsional stiffness (TS) values were calculated from the slope of the torque-angle displacement curves and normalized against the values of the contralateral femur.

### Fracture line assessment following biomechanical testing

Following biomechanical testing, the femoral segments were manually re-aligned, then carefully placed back into the freezer after the best possible fracture reduction. Subsequently, a third CT-scan was made from the fractured bones to compare the fracture lines with possible changes in the condition of the cortical bone after reaming.

The overlaying technique was performed using the software Geomagic Control 2014.3.0 (3D Systems 2016), following the approach of Gehweiler et al. (2021) [13]. The fracture starting point for each bone was defined by manual analysis, documented by bone section and direction (as classified before), and correlated with the conditions after reaming.

### Statistical analysis

Quantitative results were presented listing the number of valid cases/values, missing cases/values (if any) and total cases/values for each variable. Data from quantitative variables were summarized using the following summary statistics: mean with 95% confidence interval (CI), median, standard deviation (SD), inter-quartile range (IQR), and minimum (min.) and maximum (max.). For visualization, box- and whisker-plots were used. Non-inferiority/equivalency margins were individually set per tested characteristics if non-inferiority was defined as the statistical target (biomechanics: -20%, cortical thickness: +20%, morphological measurement and volume difference: ±20%). In front of any hypothesis testing, normality assessments were performed (Shapiro-Wilk [SW] test). In case of deviation from assumptions (e.g., normality), non-parametric methods were used for analysis. A quantitative comparison of two independent groups with the target of superiority of the ARA group were assessed using either the parametric two-independent-groups student’s t-test (if SW *p* > 0.05) or the non-parametric Mann-Whitney U Test (if SW *p* ≤ 0.05). For a quantitative comparison of two independent groups with the target of equivalency, the two-one-sided T-test (TOST) was used (if SW *p* > 0.05). To quantitatively compare the two independent groups under a non-inferiority hypothesis, the two-one-sided sign-test (TOSS) was applied, ignoring the irrelevant limit (if SW *p* ≤ 0.05). All hypothesis tests (including SW test) were conducted two-sided, performed at the 5% (0.05) significance level. Statistical analyses were performed using IBM SPSS Statistics (version 27; Armonk, NY, USA).

## Results

### Mechanical bending testing RIA 2 system and standard BixCut system reamers

Bending tests of the different test devices showed a linear bending load curve for all test samples. The bending stiffness of the BixCut System was significantly lower than the bending stiffness of the RIA 2 system (*p* = 0.004, Table 1).

### Three-dimensional geometry of cortical bone before and after harvesting

The long axis values, the ROC, and the isthmus diameter of all experimental bones were analyzed, and equivalency for the two groups was shown (Table 2).

The lowest cortical wall thicknesses after reaming in the RIA 2 group were found medially in sections 4, 5 and 6, with the minimum in section 5. After reaming the lowest cortical wall thicknesses in the ARA group were found medially in sections 4 to 6 and the minimum was found in section 6. No difference in the minimum cortical wall thickness between the groups could be found (Table 3).

**Fig. 2:**
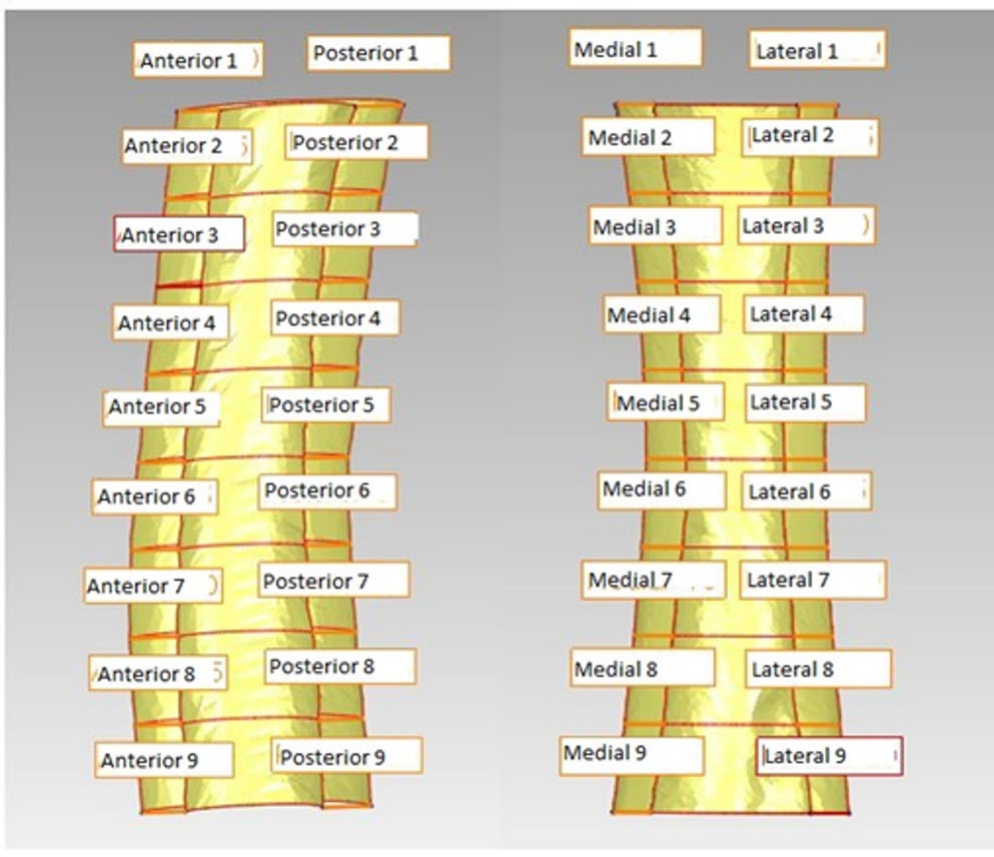
Difference in cortical bone thickness in a pre- and postoperative comparison in the transverse plane, divided into a total of 9 sections

Compared to the lateral, posterior, and anterior bone walls, the medial cortex shows the greatest reduction in cortical bone in the preoperative and postoperative comparisons. Figure 2 illustrates the decrease in cortex thickness per quartile for all transversal sections, comparing the two groups.

The lowest cortical wall thicknesses in sections 4, 5, and 6 (Table 3) are coherent with the findings of maximum difference in cortical wall thickness, which was localized in the medial direction of sections 4–6 for both groups (Table 4). Due to the high variability of the model, the data provide insufficient evidence to conclude that the population central tendency of the ARA group is non-inferior to the population central tendency of the RIA 2 group within the predefined acceptable difference and preferred direction.

### Biomechanical testing of femora ex vivo

**Fig. 3:**
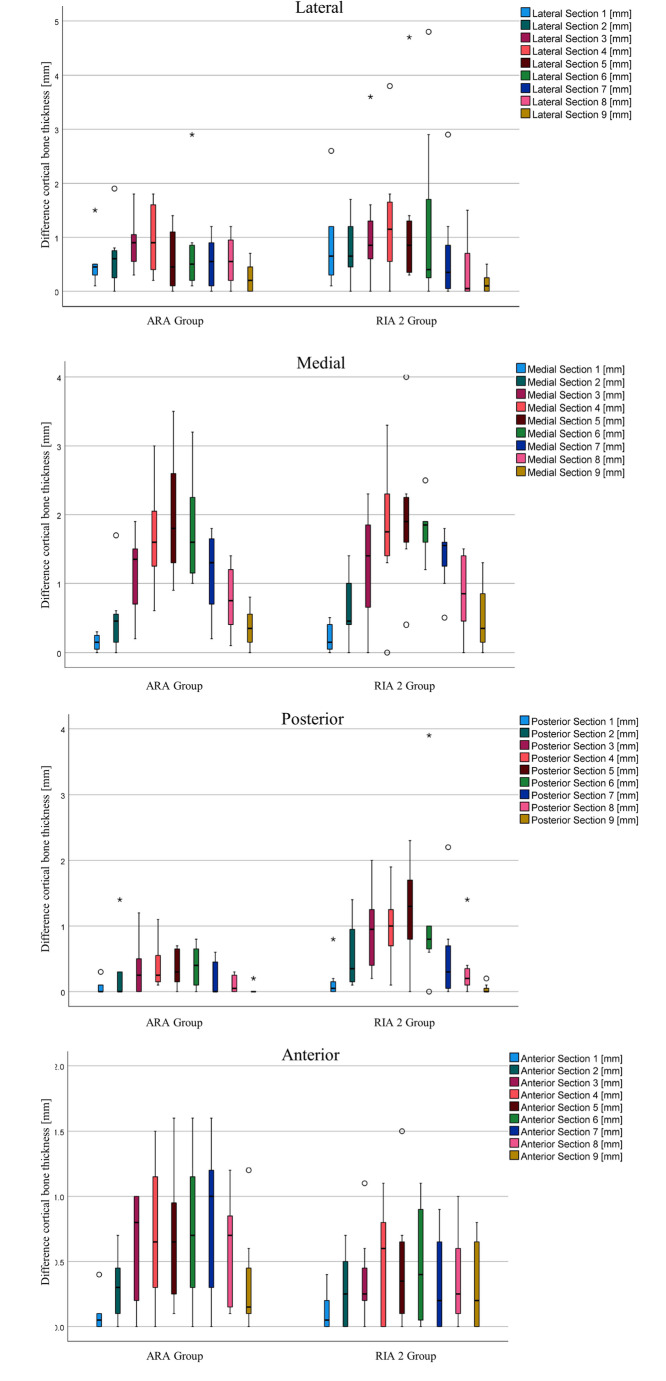
Relative stiffness and relative maximum torque comparing the operated and non-operated (contralateral) femur, green line: mean of the reference group, red line: mean of the reference group minus the non-inferiority margin (20%)

Postoperatively, frozen non-operated and operated sheep femora were thawed overnight at room temperature and underwent biomechanical testing. The evidence of the resulting data is inconclusive to assess non-inferiority between the experimental groups regarding relative stiffness (RIA 2 group: median 74.5%, IQR 35%; ARA group median 84%, IQR 38%) and maximum torque (RIA 2 group: median 22.91%, IQR 42.53; ARA group: 86.49%, IQR 72.01). Due to the high variability, more than 25% of individual data points fell below the non-inferiority margin, thus, the statistical hypothesis testing is not meaningful as the test already failed its objective (Fig. 3).

### Fracture line assessment following biomechanical testing

Fracture lines were identified on CT images in the ARA group (*n* = 8 bones) and in the RIA group (*n* = 7 bones). In one bone of the RIA group, no fracture line was identified. The analysis revealed that in the majority of cases, fractures initiated medially—between sections 3 and 7 (ARA group: *n* = 3/8, RIA 2 group: *n* = 1/7) or at sites where previous reaming had created holes (ARA group: *n* = 2/8, RIA 2 group: *n* = 4/7), with several additional fractures occurring in the anterior or lateral bone regions. Notably, most femora exhibited their lowest cortical wall thickness on the medial side, indicating that substantial medial cortical thinning from both test and control devices likely weakened bone stability in this area, increasing fracture susceptibility (Table 5).

## Discussion

The RIA 2 system has been launched in 2019 as a second-generation device applying the RIA technique for intramedullary bone graft harvesting [9]. Clinical data on the RIA 2 system regarding perioperative complications are not yet published, however, for its predecessor (RIA system), it can be concluded that procedures with very few iatrogenic fractures and intraoperative cortex weakening with eccentric reaming have been reported [7]. Therefore, any new concept applying intraoperative reaming for bone graft harvesting requires in-depth assessment of reaming centricity because eccentricity is an essential factor for weakening the bone [20]. In addition to the reaming diameter and the resulting residual cortical thickness, it is therefore also important to know whether the inner cortex is removed evenly (centric reaming) or asymmetrically (eccentric reaming) during reaming [13, 21].

Several studies have investigated the biomechanical properties of human cadaver samples after bone graft harvesting [13, 20, 22, 23]. In the development phase of new concepts for intramedullary harvesting devices, however, it is important to start much earlier, i.e., already in the preclinical in vivo phase, and to develop standardized methods for this stage of medical product development. We were able to successfully apply analysis methods previously used for human samples [13] to sheep femora and reliably obtain data. This approach also lays the foundation for future large animal in vivo studies that focus on new concepts of intramedullary bone graft harvesting and want to use validated analysis methods for the reaming trajectory early in the device development phase. The subdivision of the bone into sections was chosen to generate comparable measurements, even if minor differences in the total length of the bone occurred. This has proven to be a useful method in the analysis of the 3D geometry of bones, among others, in the previously referenced study on the reaming diameter with the RIA system [13].

The results of the analysis show that the RIA 2 and ARA group femora were well suited for comparing the reaming performance of two different systems due to their significant morphological similarity. In comparison with the measurements before reaming, the thinnest wall thickness after reaming was found in the middle part of the examination area, and in both groups an eccentric reaming behavior toward the medial side could be observed in the area of the 4th, 5th, and 6th sections. The cortical thinning analysis showed that the impact of the different reamer devices on the cortical wall thickness was not different. This indicates that the reaming performance of the test device in a one-to-one comparison to the reference device competes with its reaming rate along the length of the shaft for the orientation in the bone. Our results also confirm the recently published finding that the reduction in cortical bone volume in the same area, as examined in this study, is similar in the RIA 2 and ARA groups [9]. However, the reaming rate indicates an eccentric reaming process, showing that most material is removed medially in the middle part of the analysis area. As reported by several sources, eccentric reaming is a source of danger to the bone stability of the donor site [20, 24–27]. Therefore, with both the RIA 2 system and ARA concept care should be taken to ensure centric reaming, although initially no tendency could be determined as to which device is more prone to eccentric reaming. Analytics showed, however, that the bending stiffness of the BixCut device is significantly lower than the bending stiffness of the RIA 2 device.

To the best of our knowledge, the work of Gehweiler et al. (2021) [13] is the only other work that could be found investigating the 3D geometry of the reaming performance in the bone. There, the reaming was conducted using human cadaver bones, and the focus was on the comparison of the influence of the reamer head diameter, showing that a higher reamer head diameter leads to a higher decrease in cortical wall thickness and a higher harvest volume. Also, the study showed that the highest decreases in wall thickness occurred medially in the central part of the bone length for all reamer head sizes. Thus, their results match the results of the work presented here.

In the current study, it is the first time that the mechanical behavior of a reamed, unstabilized femur in sheep is described. Interestingly, while Gehweiler et al. (2021) [13] made entry point at the greater trochanter, our study utilized the intertrochanteric (piriformis) fossa; however, no differences in biomechanical stability or fracture line trajectory were observed. This means that the use of the sheep femur does indeed have a translational value in comparison to the human femur, as no difference in biomechanical stability was found in the human femur in a cadaveric study using different entry points (trochanteric versus piriformis fossa versus retrograde) [28]. In our protocol, the first reamer head size adapted to the isthmus diameter (starting 2 mm smaller compared to the isthmus diameter) was initially used for all test femurs and the reamer head sizes were then increased accordingly until 1–2 mm of residual cortex was still visible under standard X-ray technique. We therefore adhered to the clinical recommendation to adapt the size of the reamer head to the patient’s femur and used the possibilities of the RIA 2 system and BixCut reamers to increase the size of the reamer head. In contrast to previous studies [13, 28] that only used one reamer head size, this allowed us to better simulate the clinical situation and achieve more translationally valuable results.

## Limitations

This study has similar limitations to all in vivo studies, as it is not able to fully simulate the clinical situation. However, the described analysis method and comparison concept can be applied to human bones in the future. While this work provides a first impression of the effects of using the RIA 2 system and ARA concept (including the use of BixCut reamers) on bone geometry as well as guidance for the analysis steps, application to human bone will be the next objective in the geometric evaluation of intramedullary reaming performance. In the clinical use of the RIA system, only 1–3 mm of cortical bone is removed during the harvesting process [29], whereas in this study, as recently published in detail [9], more aggressive reaming was performed until only 1–2 mm of cortex remained. Therefore, based on this study, it cannot be conclusively predicted to what extent the RIA 2 system and the ARA concept ensure biomechanical stability; this needs to be addressed in follow-up studies.

## Conclusions

In this preclinical in vivo comparison, both systems demonstrated comparable reaming behavior with respect to cortical bone removal and geometric alteration of the femoral shaft. Although the RIA 2 drive shaft showed higher bending stiffness, both systems produced similar reaming characteristics and patterns of cortical thinning, with predominant medial reduction in the mid-diaphyseal region. Three-dimensional geometric assessment and ex vivo biomechanical testing showed no relevant differences in residual cortical thickness or torsional stability between groups. Fracture initiation patterns were comparable and were mainly associated with regions of maximal medial cortical thinning, indicating a similar structural response to reaming. Notably, the analysis of postsurgical cortical thickness reduction and the tendency of these weakened regions to fail under torsional loading provided valuable insight into the impact of reaming-induced cortical thinning on bone stability. From the results of the one-to-one comparison, it can be concluded that the RIA 2 system and the ARA concept, including utilization of BixCut reamers, provide equivalent results in reaming functionality.

**Table 1:** Bending stiffness of RIA 2 system and BixCut reamer shafts

Scores		Classification Groups		*p*-value
		BixCut System	RIA 2 System	
N		6	6	-
Bending Stiffness [N/mm]	Mean (± SD)	17.82 (± 2.2)	63.35 (± 1.1)	*0.004* ^*^
	Median (IQR)	17.8 (4.2)	63.6 (1.8)	
	Range (Min.–Max.)	3.1 (61.7–64.8)	70 (10–80)	

* Mann-Whitney U

SD: Standard Deviation

IQR: Interquartile Range

Underlined results are significant at the 5% level (0.05)

**Table 2:** Morphological parameter of bones from ARA and RIA 2 group

Scores		Classification Groups		*p*-value
		ARA	RIA 2	
N		8	8	-
Long axis [mm]	Mean (± SD)	190.11 (± 4.5)	188.11 (± 11.0)	< 0.001**
	Median (IQR)	190.45 (5)	186.75 (17)	
	Range (Min.–Max.)	15.2 (181.3-196.5)	33.6 (171.9-105.5)	
ROC [mm]	Mean (± SD)	255.78 (± 16.3)	277.21 (± 36.9)	0.001**
	Median (IQR)	257.8 (21.5)	269.05 (48.2)	
	Range (Min.–Max.)	51.24 (235.5-286.7)	117.50 (231.7-349.2)	
Isthmus diameter [mm]	Mean (± SD)	11.49 (± 0.8)	10.8 (± 1.2)	0.001**
	Median (IQR)	11.55 (1.5)	10.95 (0.8)	
	Range (Min.–Max.)	2.4 (10.1–12.5)	4.2 (8.3–12.5)	

** TOST

SD: Standard Deviation

IQR: Interquartile Range

Underlined results are significant at the 5% level (0.05)

**Table 3:** Lowest cortical wall thickness comparing RIA 2 group with ARA group

Scores		Classification Groups		*p*-value
		ARA	RIA 2	
N		8	8	-
Medial section 4, post-op cortical wall thickness [mm]	Mean (± SD)	1.94 (± 0.51)	1.84 (± 0.88)	0.785^+^
	Median (IQR)	2.0 (0.9)	1.8 (0.7)	
Medial section 5, post-op cortical wall thickness [mm]	Mean (± SD)	1.93 (± 0.29)	1.62 (± 0.88)	0.370^+^
	Median (IQR)	2.0 (0.5)	1.55 (1.3)	
Medial section 6, post-op cortical wall thickness [mm]	Mean (± SD)	1.81 (± 0.56)	1.79 (± 0.57)	0.945^+^
	Median (IQR)	1.9 (0.8)	1.75 (1.0)	

^+^Two-independent samples t-test

SD: Standard Deviation

IQR: Interquartile Range

Underlined results are significant at the 5% level (0.05)

**Table 4:** Difference after bone removal in medial wall thickness comparing both experimental groups

Scores		Classification Groups		*p*-value
		ARA	RIA 2	
Valid N		8	8	-
Medial section 1, difference cortical wall thickness [mm]	Mean (± SD)	0.15 (± 0.12)	0.21 (± 0.2)	0.289^++^
	Median (IQR)	0.15 (0.3)	0.15 (0.4)	
Medial section 2, difference cortical thickness [mm]	Mean (± SD)	0.5 (± 0.53)	0.64 (± 0.46)	0.07^++^
	Median (IQR)	0.45 (0.5)	0.45 (0.7)	
Medial section 3, difference cortical thickness [mm]	Mean (± SD)	1.15 (± 0.56)	1.26 (± 0.8)	0.07^++^
	Median (IQR)	1.35 (0.8)	1.4 (1.5)	
Medial section 4, difference cortical thickness [mm]	Mean (± SD)	1.68 (± 0.53)	1.78 (± 0.96)	0.289^++^
	Median (IQR)	1.6 (0.9)	1.75 (1.1)	
Medial section 5, difference cortical thickness [mm]	Mean (± SD)	1.98 (± 0.87)	1.99 (± 1.01)	0.289^++^
	Median (IQR)	1.8 (1.7)	1.9 (0.7)	
Medial section 6, difference cortical thickness [mm]	Mean (± SD)	1.775 (± 0.79)	1.8 (± 0.37)	0.289^++^
	Median (IQR)	1.6 (1.3)	1.85 (0.3)	
Medial section 7, difference cortical thickness [mm]	Mean (± SD)	1.16 (± 0.58)	1.39 (± 0.43)	0.289^++^
	Median (IQR)	1.3 (1.0)	1.555 (0.5)	
Medial section 8, difference cortical thickness [mm]	Mean (± SD)	0.78 (± 0.47)	0.86 (± 0.57)	0.289^++^
	Median (IQR)	0.75 (0.9)	0.85 (1.1)	
Medial section 9, difference cortical thickness [mm]	Mean (± SD)	0.36 (± 0.28)	0.5 (± 0.5)	0.125^++^
	Median (IQR)	0.35 (0.5)	0.35 (1.0)	

^++^ TOSS

SD: Standard Deviation

IQR: Interquartile Range

Underlined results are significant at the 5% level (0.05)

**Table 5:** Fracture line starting points assessment comparing the ARA with the RIA 2 group following biomechanical testing

Specimen no	Lowest cortical wall thickness	Fracture start
*ARA group*		
1	Medial section 4	Anterior section 5
2	Medial section 5	Medial section 4
3	Hole posteromedial section 5	Posteromedial hole sections 5–7
4	Medial section 4	Medial section 6
5	Lateral section 1	Medial between sections 6 to 7
6	Hole medial section 5	Medial section 5
7	Medial section 5	Posterolateral section 9
8	Hole medial sections 4–6	Anterior section 6
*RIA 2 group*		
1	Lateral hole section 1	Lateral hole section 1
2	Medial section 5	Medial section 5
3	Medial sections 5/6	Not identifiable
4	Medial section 5	Lateral sections 7
5	Lateral section 1	Anterior section 8/9, lateral sections 5
6	Hole medial section 4	Medial section 4
7	Hole medial section 5	Medial section 4
8	Hole medial sections 3–6	Medial section 3

## Supplementary Information

Below is the link to the electronic supplementary material.

Supplementary Material 1

Supplementary Material 2

## Data Availability

The data that support the findings of this study are available from the corresponding authors upon reasonable request.
