# Supplementary material for: Comparative assessment of the reaming characteristics of two different technologies for intramedullary bone graft harvesting
Source: Arch Orthop Trauma Surg. 2026 Apr 9;146(1):138. doi: 10.1007/s00402-026-06270-y (PMC13065597; doi:10.1007/s00402-026-06270-y)

Comparative assessment of the reaming characteristics of two different technologies for intramedullary bone graft harvesting

Simone Guttau^1,2,^*^,†^; Merle Lange^1,^*; Ronja Finze^3^; Claudia Beimel^1^; Siamak Saifzadeh^4,7^; Jonathan Gospos^8,9^; Marie-Luise Wille^4,8,9^; Philipp Kobbe^2,5^; Markus Laubach^4,6,†^

*shared first authorship

^1^ Stryker Trauma GmbH, Schönkirchen, Germany.

^2^ Department for Trauma and Reconstructive Surgery, University Hospital of the Martin Luther University Halle, Halle (Saale), Germany

^3^ Department of Hand, Plastic, and Reconstructive Surgery, Microsurgery, Burn Center, BG Trauma Centre Ludwigshafen, University of Heidelberg, Ludwigshafen, Germany

^4^ Australian Research Council (ARC) Training Centre for Multiscale 3D Imaging, Modelling, and Manufacturing (M3D Innovation), Queensland University of Technology, Brisbane, QLD 4000, Australia

^5^ Department for Trauma and Reconstructive Surgery, BG Hospital Bergmannstrost Halle, Halle (Saale), Germany.

^6^ Department of Orthopaedics and Trauma Surgery, Musculoskeletal University Center Munich (MUM), LMU University Hospital, LMU Munich, Munich, Germany

^7^ Medical Engineering Research Facility, Queensland University of Technology, Chermside, QLD 4032, Australia.

^8^Max Planck Queensland Centre (MPQC) for the Materials Science of Extracellular Matrices, Queensland University of Technology (QUT), Brisbane, QLD 4000, Australia.

^9^Centre for Biomedical Technologies, School of Mechanical, Medical and Process Engineering, Queensland University of Technology, Brisbane, QLD 4000, Australia.

*shared first authorship

†Corresponding authors: **Simone Guttau**, Stryker Trauma GmbH, Schönkirchen, Germany, E-mail: Simone.Guttau@stryker.com; **Markus Laubach**, Department of Orthopaedics and Trauma Surgery, Musculoskeletal University Center Munich (MUM), LMU University Hospital, LMU Munich, Munich, Germany, E-mail: Markus.Laubach@med.uni-muenchen.de

# Supplementary data

# Supplementary Figure 1

Experimental bending test setup


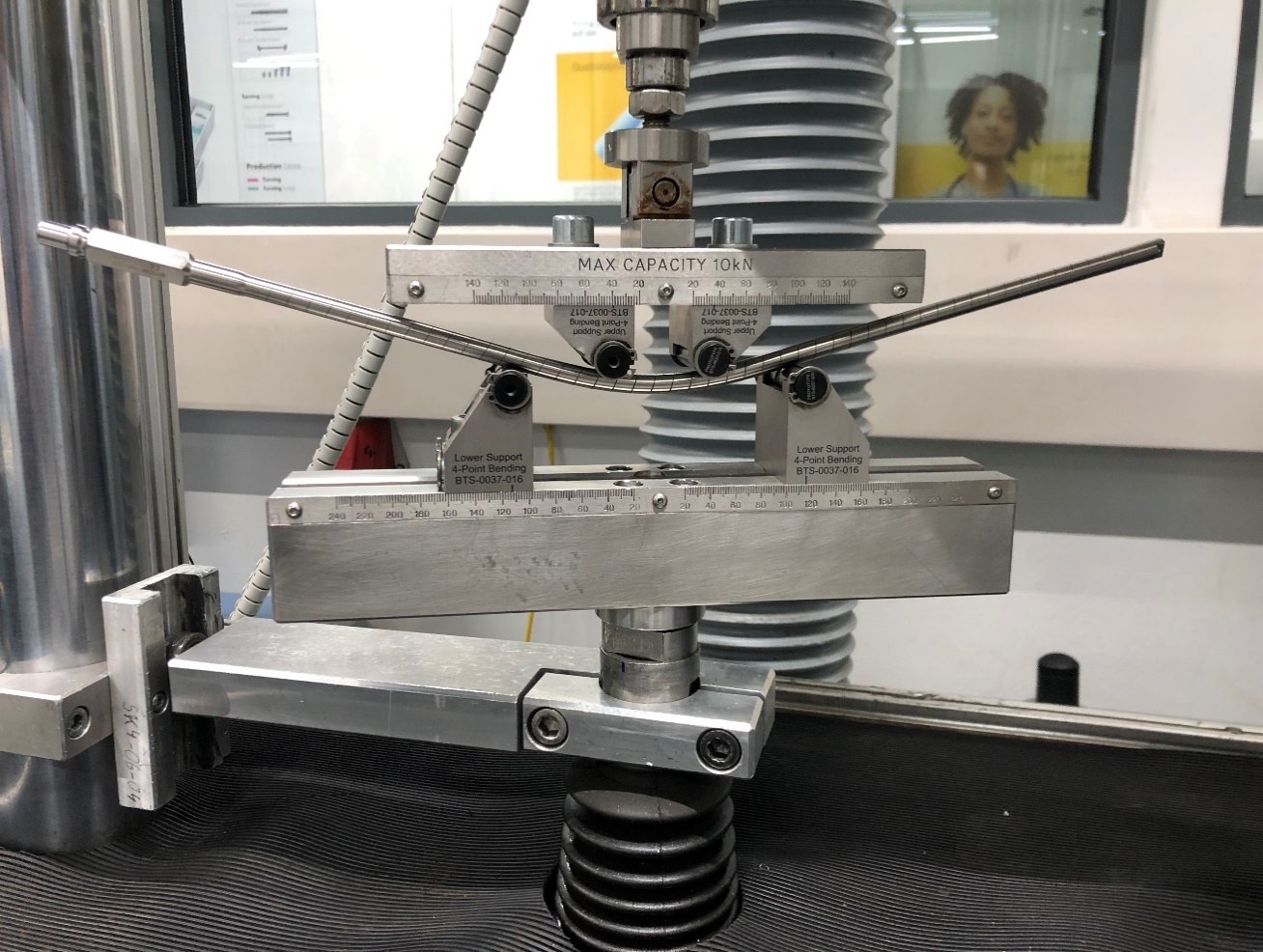


114mm

38mm

# Supplementary Figure 2

Setup biomechanical testing
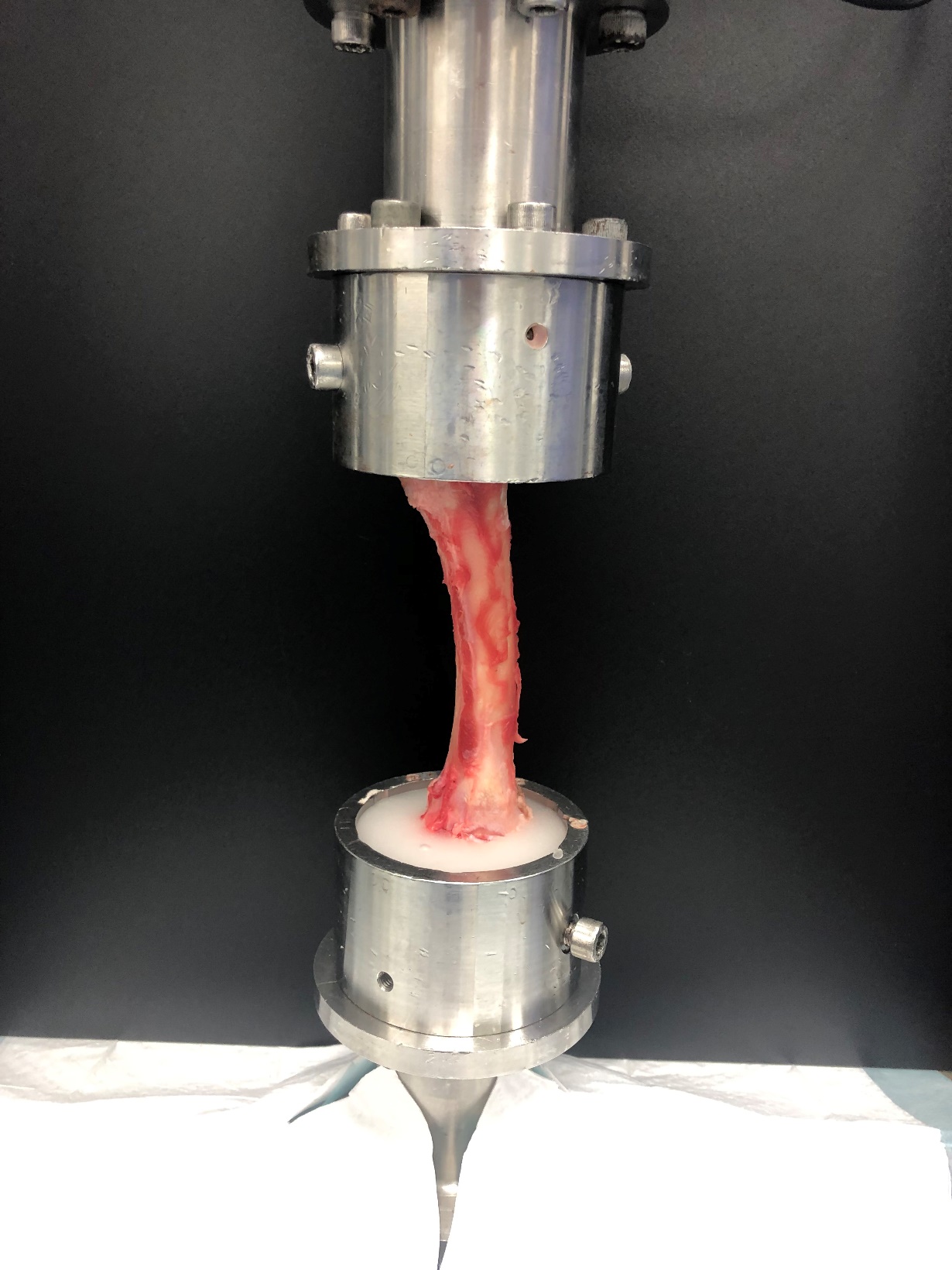

Supplement: Supplementary file 1 — Supplementary Material 1 [file 402_2026_6270_MOESM1_ESM.docx]
